# Relating Infant Fixations to Adult Cortical Activation Patterns Using the Natural Scenes Dataset

**DOI:** 10.1111/desc.70076

**Published:** 2025-09-19

**Authors:** Brianna K. Hunter, John E. Kiat, Steven J. Luck, Lisa M. Oakes

**Affiliations:** ^1^ Center for Mind and Brain University of California Davis California USA; ^2^ Department of Psychology University of California Davis California USA

**Keywords:** dorsal visual stream, infancy, Natural Scenes Dataset (NSD), representational similarity analysis (RSA), ventral visual stream, visual attention

## Abstract

Visual attention develops rapidly across the first postnatal year, from reflexive eye movements driven by low‐level stimulus properties to increasingly voluntary eye movements influenced by higher‐order factors. To test the hypothesis that development reflects guidance by increasingly abstract features, we used representational similarity analysis to evaluate the representational link between gaze patterns (*N* = 47 5–7‐month‐old infants, *N* = 46 10–12‐month old infants, *N* = 45 adults) and measurements of fMRI cortical activity patterns from adult participants as they viewed scenes from the Natural Scenes Dataset. We found that similarities across scenes for fixation patterns and neural activity patterns were significantly related only for low‐level visual regions in younger infants but were related to mid‐level regions in older infants and adults. These results support the hypothesis that, over development, visual attention shifts from being driven by the kinds of simple features represented in early visual cortex to being driven by the kinds of more abstract features found in mid‐level areas of adult visual cortex. A video abstract of this article can be viewed at https://www.youtube.com/watch?v=WkIV9QB0Uq8.

**Summary:**

- Prior research demonstrates that infant eye movements are initially driven by low‐level stimulus properties but become increasingly adult‐like and controlled by more abstract representations.
- We evaluated the link between cortical activity patterns in adults (from the Natural Scenes Dataset) and gaze patterns in infants and adults viewing naturalistic scenes.
- Fixation patterns were related only to low‐level ventral stream regions in younger infants but to both low‐ and mid‐level regions in older infants and adults.
- These results support the hypothesis that visual attention development reflects guidance by increasingly abstract features like those coded by higher‐order areas of adult visual cortex.

## 1 Introduction

The factors guiding where infants look shift across the first postnatal year, from being biased toward physically salient items to becoming increasingly directed toward meaningful parts of the environment (Colombo 2001; Frank et al. 2009; Kwon et al. 2016). This development suggests that infants’ attention is increasingly guided by more abstract representations of the visual world. That is, where infants look becomes influenced by factors beyond basic visual features (e.g., edges, orientation), such as object shape or semantic content. However, the exact nature of these representations is not well understood. Here, we operationalized different levels of abstraction using high‐resolution fMRI data obtained from low‐, mid‐, and high‐level regions of the ventral stream in adults, available in the Natural Scenes Dataset (NSD) (Allen et al. 2022). We then examined how these patterns of adult neural activity predicted gaze patterns of younger infants, older infants, and adults as they viewed scenes from this dataset.

The study of infant visual attention has historically distinguished between two processes (Cohen 1973). The first, *attention‐getting*, refers to the mechanisms that determine where infants look, particularly when viewing multiple competing stimuli. The second, *attention‐holding*, refers to the mechanisms that determine how long infants look at a given object or scene region. Although these two attention processes work together to support learning (Fisher 2019), they have historically been thought to be influenced by different features of the environment. For instance, when presented with two checkerboards, the sizes of the checks influenced *where* infants looked, but the number of checks influenced *how long* infants looked (Brennan et al. 1966). Decades of research converged with this notion, finding that when infants are presented with one or two stimuli at a time, attention‐getting processes are sensitive to the low‐level physical characteristics of stimuli (e.g., size), whereas attention‐holding processes are influenced by higher‐level cognitive factors such as recognition memory (Rose et al. 1982), categorization (Oakes and Ribar 2005), and the intention of actors (Woodward 1998). This has led to an extensive literature examining infants’ developing representation of visual information, relying primarily on how long infants look at visual stimuli (Ayzenberg and Behrmann 2023; Kellman and Arterberry 2000).

However, adult attention researchers have largely come to agreement that a dichotomous view of attention processes (e.g., attention‐getting vs. attention‐holding) is overly simplistic (Anderson 2024; Awh et al. 2012; Luck et al. 2021). Rather, where an individual looks is guided by a priority map that appears to integrate multiple factors—such as physical salience, prior learning, motivational relevance, and task goals—to dynamically compute the attentional priority of each location in the visual field (Todd and Manaligod 2018). Infant research has been slower to adopt this view, in part due to the delayed availability of automatic eye‐tracking equipment and algorithms designed for collecting data from infants (Aslin and McMurray 2004; Oakes 2012). These technological advances now allow researchers to precisely measure the spatial distribution of infants’ gaze across more complex arrays or naturalistic scenes (e.g., photographs of everyday places such as kitchens, landscapes, and parks) that contain many items for infants to look at, similar to the visually rich real‐world. A growing body of work has therefore considered how both low‐ and high‐level stimulus features influence where infants look when viewing more complex displays.

Infants’ fixation locations can be predicted by models of physical salience inspired by the properties of V1 and V2 of primate visual cortex, with the spatial distribution of younger infants’ fixations being more consistent with these salience models compared to older infants (Mahdi et al. 2018; Pomaranski et al. 2021). Moreover, some low‐level properties, such as orientation, have a stronger effect on where infants look compared to other low‐level properties, such as color (Hunter et al. 2024). Going beyond low‐level features, Oakes et al. (2024) found that *meaning maps*—quantitative stimulus maps that reflect high‐level semantic informativeness of scene regions (Henderson and Hayes 2017)—became increasingly predictive of the spatial distribution of infants’ fixation locations as they viewed naturalistic scenes across the first postnatal year. These results are consistent with findings that, compared to younger infants, older infants are more likely to look towards faces, which are socially informative and highly meaningful, in visual search arrays (Kwon et al. 2016) and complex dynamic scenes (Frank et al. 2009).

Although this prior research has demonstrated that where infants look (i.e., attention‐getting) becomes increasingly influenced by abstract features over development, much less is known about how infant gaze is guided by the kinds of increasingly abstract features coded by successive regions along the ventral stream. It appears that infants’ attention‐holding is increasingly related to the types of feature abstraction along the ventral visual processing stream, at least when viewing isolated objects. Spriet et al. (2022) compared activation of adults’ visual cortex to the overall looking times of 4‐, 10‐, and 19‐month‐old infants as they viewed isolated objects against a grey background. The relation between infants’ looking duration and the adult visual cortex increased with age, suggesting that infants' overall engagement with objects was increasingly related to more complex visual processes. Kiat et al. (2022) provided complementary evidence about attention‐getting, showing that *where* infants look as they view naturalistic scenes also becomes increasingly related to higher level cortical activity. Specifically, Kiat and colleagues related 4–12‐month‐old infants’ spatial distribution of fixations (i.e., where they looked) to AlexNet (Krizhevsky et al. 2012, a convolutional neural network model inspired by the primate ventral pathway). AlexNet contains five topographically mapped convolutional layers that extract increasingly abstract visual information, similar to how visual input is transformed as it makes its way up the ventral visual pathway from V1 through inferotemporal cortex. Using *representational similarity analysis* (RSA), Kiat and colleagues found that lower‐level layers of AlexNet were more related to the gaze patterns of younger infants, with higher‐level layers being more related to the gaze patterns of older infants. Thus, the existing evidence is consistent with the hypothesis that, across the first postnatal year, gaze becomes increasingly controlled by the kinds of abstract representations found in mid‐ and high‐level regions of the ventral stream.

The present study extended this work by relating infant gaze patterns to adult visual cortex representations while they viewed naturalistic scenes. We obtained the visual cortex data from the NSD (Allen et al. 2022), which contains fMRI data recorded from adults while they viewed thousands of scenes. We then recorded eye tracking data from infants while they looked at a subset of the scenes from this existing dataset. Using the RSA approach adopted by Kiat et al. (2022), we examined the extent to which the spatial distribution of infants’ attention across scenes was related to the cortical representations in different areas of visual cortex and whether this relationship varied with infant age. Importantly, the NSD is organized into predefined regions of interest (ROIs), some of which correspond to low‐, mid‐, and high‐level regions of the ventral visual stream that are roughly analogous to the AlexNet layers examined by Kiat et al. This allowed us to examine how the infants’ patterns of fixations for different scenes were related to adult visual cortical activity across voxels produced by those scenes.

The present study tested the specific hypothesis that, over development, attention‐getting becomes progressively more controlled by the type of increasing abstraction observed in adult visual cortex as information travels up the ventral processing stream. We predicted that the spatial distribution of younger infants’ fixation would be linked most closely with adult neural activity patterns in lower‐level visual cortex, whereas the spatial distribution of older infants’ fixation would be linked more closely with adult activity patterns in higher‐level areas of the ventral stream. However, we expected that our approach of relating the spatial distribution of gaze to fMRI activity patterns would work best in ROIs with strong topographic mapping in which the spatial location of visual input is preserved across voxels. Therefore, effects among older infants may be limited to mid‐level regions only.

Our focus on the ventral stream may seem surprising given that areas such as the frontal eye fields and posterior parietal cortex play a much more direct role in directing individual eye movements. However, prior research shows that the *pattern* of fixations across a natural scene in both older infants and adults is strongly influenced by the kinds of relatively abstract features coded by mid‐ and high‐level areas of the ventral stream (Hayes and Henderson 2021; Henderson and Hayes 2017; Kiat et al. 2022; Oakes et al. 2024). Moreover, participants in the NSD study were required to keep their gaze fixed at the center of the display while viewing the scenes, which presumably reduced fMRI activity related to shifts of overt attention. This might minimize our ability to observe a strong relationship between oculomotor structures in the fMRI data and the eye movement patterns. However, we did conduct exploratory analyses on two dorsal stream ROIs given that covert orienting of attention develops across the first postnatal year (Richards 2005; Ross‐Sheehy et al. 2015).

## 2 Method

### 2.1 Participants

*Infants*. The final sample included 93 infants tested in Davis, California, between April 18, 2023, and July 25, 2024. Infants were recruited from the suburban and urban regions surrounding Davis and Sacramento, California in two age groups as follows: 47 5–7‐month‐old infants (28 girls, 19 boys; *M* = 6 months, 0 days, SD = 18.0 days; 154–213 days) and 46 10–12‐month‐old infants (25 girls, 21 boys; *M* = 11 months, 7.50 days, SD = 13.5 days, 315–366 days). Sample size was determined a priori by simulating repeated measures data using population values based on the smallest effect size of interest (Cohen's *f* = 0.1; Anvari and Lakens 2021). Our simulations indicated that a final sample of 80 infants (40 per age group) would achieve a power of 0.8.

All infants were full‐term, typically developing, and reflected diverse backgrounds consistent with the local demographics. Fifty‐three were White (16 Hispanic), 10 were Asian or Asian American (0 Hispanic), three were Black or African American (two Hispanic), 23 were Multiracial or other (nine Hispanic), and four had no race reported (four Hispanic). Eighty‐nine mothers of infants completed some college, with 72 of these mothers attaining a bachelor's degree or higher. Sixty‐six families had an annual income greater than $100,000, 14 between $50,000 and $99,999, 10 less than $50,000, and three families did not report income.

We tested 29 additional infants but excluded them from final analyses due to fussiness (*n* = 7), equipment error (*n* = 2), parental interference (*n* = 2), failure to successfully calibrate (*n* = 3), or not contributing a sufficient number of trials (*n* = 15). Full demographics for the excluded infants can be found at https://osf.io/juw78/. Families were recruited from the California State Office of Vital Records and compensated with a completion certificate and a small toy.

*Adults*. Because the adults in the original NSD study were instructed not to make any eye movements, we collected eye tracking data from a new set of 45 adults (*M*_age_ = 20.27 years, SD = 1.51) while they freely viewed the scenes. These participants were tested in Davis, California, between April 29, 2025, and June 5, 2025. All of the adult participants were college students; 29 identified as female, 13 identified as male, one identified as nonbinary or a third gender, and two did not report gender identity. All had normal or corrected to normal vision and were representative of a diverse university campus: 10 were White (four Hispanic), 28 were Asian (zero Hispanic), one was Black (not Hispanic), one was Native Hawaiian/Pacific Islander (not Hispanic), and five did not report race (five Hispanic). They received course credit for participating. We did not obtain information about income, but 41% of new students at the University of California, Davis in 2025 were first‐generation college students and 39% were recipients of Pell Grants for low‐income students (Easley 2025), suggesting a broad range of income levels.

### 2.2 Stimuli

Stimuli were presented on an LCD video monitor (ASUS VG248QE) with a 1920 × 1080 resolution (53 × 30 cm) at a viewing distance of approximately 60 cm. Each stimulus was a 1000 × 1000 pixel scene (subtending 26° × 26° of visual angle), centered on the display. The remainder of the display was gray.

**FIGURE 1:**
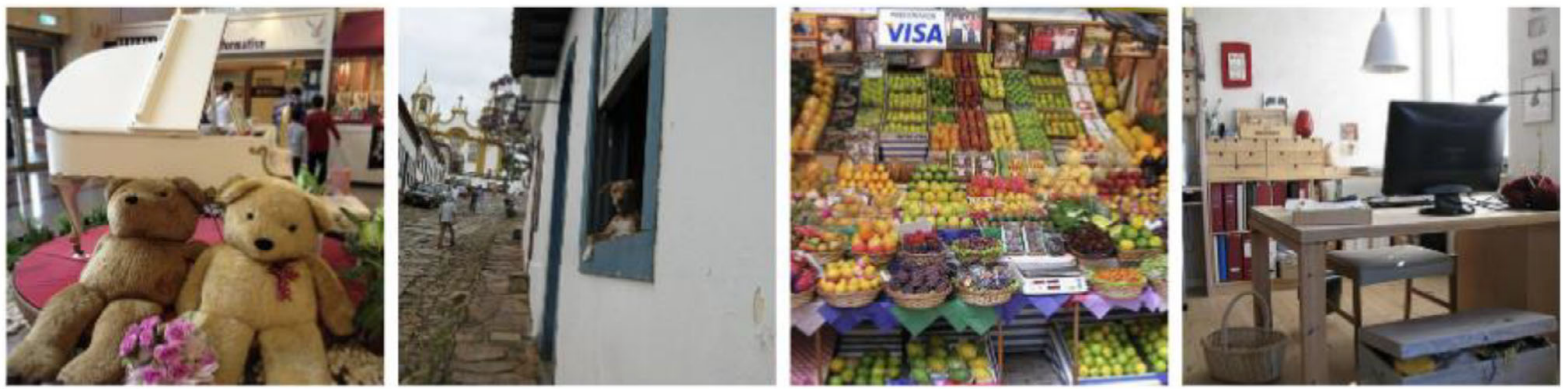
Examples of stimuli chosen from the NSD.

We selected 78 scenes (of the available 73,000 scenes) from the NSD, which were originally taken from the COCO image set (Lin et al. 2015). We selected images that (1) contained infant‐friendly content (e.g., no alcohol or adult themes) and (2) were viewed three times by all eight adult NSD participants while in the MRI scanner. The images included content such as landscapes, pets, living spaces, offices, and parks (see Figure 1 for examples; see all 78 images at https://osf.io/juw78/).

### 2.3 Apparatus and Procedure

*Infants*. We measured infants’ fixations using an SR Research EyeLink1000 Plus eye tracker with a sampling rate of 1000 Hz. Infant participants sat in a high chair or a caregiver's lap. The eye tracker was affixed to the monitor, and both were on a moveable arm that could be adjusted. Experimenter Builder, software provided by SR‐Research, controlled the stimulus presentation. Caregivers wore opaque glasses to obstruct their view of the stimuli during the session.

We used a standard 5‐point calibration procedure. A looming circle appeared in each target location, moving once the experimenter accepted a fixation at that location. This was repeated to validate calibration. If calibration accuracy was poor (e.g., average systematic error > 2° overall, or one of the fixations appeared in an obvious outlier position), the procedure was repeated. The average calibration for participants included in the final analysis was good (*M* = 0.76°, SD = 0.50°).

After calibration, infants viewed up to 50 randomly selected scenes presented in a random order. Based on prior infant scene studies (e.g., Pomaranski et al. 2021; Oakes et al. 2024), we estimated that infants could complete approximately 50 trials before becoming fatigued. Data simulations suggested that presenting a greater number of scenes in a random order (and thereby increasing the total number of scenes across infants) would reduce result variability compared to presenting a set of 50 scenes in a fixed order.

Before each trial, a small cartoon with attention‐getting sounds oriented the infant to the center of the monitor; once the eye‐tracker detected 500 ms of fixation to the cartoon, a stimulus appeared in the center of the screen for 5 s, during which infants freely viewed the scene. To maintain interest, each stimulus was accompanied by classical music. This procedure continued until (a) 50 stimuli were presented, (b) the participant refused to look at the screen, or (c) the eye tracker could no longer maintain a track on the participants’ eyes (usually due to fussiness).

*Adults*. The apparatus and procedure for adult participants were nearly identical to those used for the adult samples. To mirror the testing conditions as closely as possible to the infant sample, the adults were not given any explicit instructions other than to look at the screen (i.e., “free viewing task,” commonly used in adult scene viewing studies; Itti et al. 1998). In contrast to the infant sample, which was recorded using SR‐Research's remote mode to accommodate head movement, adult participants viewed all stimuli while their heads were stabilized using a chinrest. Adult data were recorded at 500 Hz, whereas infant data were recorded at 1000 Hz.

### 2.4 Data Processing

#### 2.4.1 Eye Movement Data Processing

We used SR‐Research's default fixation detection parameters to segment fixations from saccades. Trials were excluded if (1) the participant was not looking at the center of the screen when the trial began (2.20% of all infant trials, 0% of adult trials), (2) the participant only looked at the portion of the screen that did not contain the images (0.37% of all infant trials, 0% of adult trials), (3) the participant made fewer than three total fixations (6.04% of remaining infant trials, 1.80% of adult trials), or (4) the sum of fixation durations was less than 1000 ms (3.46% of remaining infant trials, 0% of adult trials). Finally, we excluded any participants who did not contribute at least 18 trials of usable data (*n* = 15 infants, *n* = 0 adults), mirroring the exclusionary criterion reported by Kiat et al. (2022). After all exclusions, we had 3712 trials across our 93 infants and 2235 trials across the 45 adults.

We used the fixation data to create *fixation density maps* for each scene viewed by each participant. As is standard practice in the field, we excluded the first fixation on each trial (it was always in the center of the screen). Fixation density maps were created as 2D image matrices in Matlab using the approach described in Pomaranski et al. (2021), which was based on adult scene research (Henderson and Hayes 2018) and the MIT/Tuebingen Saliency Benchmark code (Judd et al. 2012). First, for each individual trial, we created a matrix of zeros corresponding to each pixel in the image (1000 × 1000). Next, we added “1” to the cells corresponding to the *X*/*Y* coordinates of each fixation within the image. To account for eye tracking error, we applied a Gaussian low‐pass filter with a circular boundary and cutoff frequency of −6 dB, corresponding to a standard deviation of approximately 7.21 pixels. Finally, the maps were normalized using Matlab's mat2gray function so that all cells in the matrix contained values ranging from 0 (the least fixated pixel) to 1 (the most fixated pixel).

#### 2.4.2 fMRI Data From the Natural Scenes Dataset (NSD)

Our primary analyses examined the correspondence between similarities in the fixation density maps that we obtained from infants and adults and similarities in the fMRI data from the preexisting NSD (full details of data collection can be found in Allen et al. 2022). The NSD data were obtained from eight right‐handed adults with normal or corrected‐to‐normal vision, no diagnosed cognitive deficits or color blindness, and an established record of producing high‐quality, low‐artifact data. During fMRI data collection, each stimulus appeared for 3 s, followed by a 1s break. Participants maintained their fixation in the center of each image and pressed a button to indicate whether they had seen the image before. Thus, the fMRI data did not reflect activity related to programming and executing eye movements, although the recorded activity may include contributions from covert shifts of attention.

**FIGURE 2:**
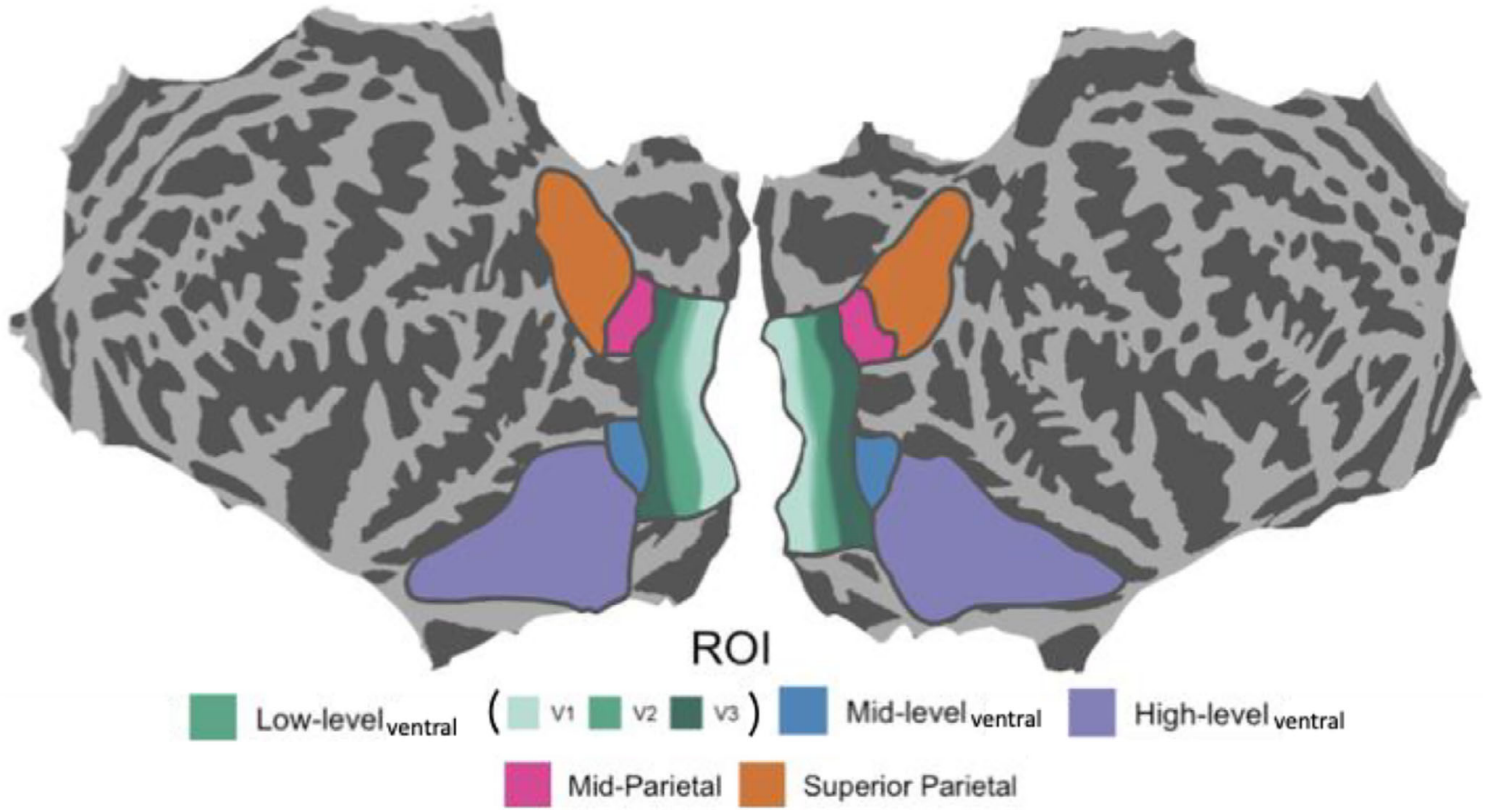
ROIs used in the analyses of the NSD dataset. Top: ventral stream analysis. ROIs originate in the “streams” NSD data, but the names of the ROIs have been modified here for ease of interpretation. The *low‐level_ventral_* ROI here was originally labeled “early” in NSD, *mid‐level_ventral_* was originally labeled “midventral” in NSD, and *high‐level_ventral_* was originally labeled “ventral” in NSD. Middle: early visual cortex analysis. V1 ROI was computed by appending the V1v and V1d ROIs in the NSD, V2 combined V2v and V2d, and V3 combined V3v and V3d. Specific borders were drawn for each NSD subject based on the prf experiment. Bottom: dorsal stream analysis. These ROIs also originated in the “streams” NSD data, but the names are modified here. *Mid‐parietal* ROI here was originally labeled “midparietal” in NSD, and *superior parietal* was originally labeled “parietal” in NSD.

We used the adult fMRI data to conduct analyses on three groups of ROIs that were evaluated in separate statistical models. First, in our primary, a priori analyses, we evaluated the three ventral visual stream ROIs (low‐level_ventral_, mid‐level_ventral_, high‐level_ventral_), which were already derived within the NSD dataset (see Figure 2). The *low‐level_ventral_* ROI included the V1v, V1d, V2v, V2d, V3v, and V3d ROIs from the Wang et al. (2015) atlas. The *mid‐level_ventral_* ROI reflected the region bordered by the inferior boundary of hV4 and the anterior boundary of the inferior occipital sulcus (IOS). Finally, the *high‐level_ventral_* ROI followed the anterior lingual sulcus (ALS), and spanned the area from the IOS to the midpoint of the occipital temporal sulcus (OTS). Thus, these three ROIs covered regions that are responsible for the processing of basic visual features (e.g., orientation) through regions that are involved in more complex visual processing, such as object recognition and scene perception.

In our second set of NSD analyses, we probed early visual cortex in the ventral stream further by disaggregating areas within the low‐level ROI (Figure 2). Specifically, the NSD includes separate ROIs for V1v, V1d, V1d, V2v, V2d, V3v, and V3d, which correspond to dorsal and ventral subdivisions of V1, V2, and V3. Instead of these regions being drawn based on the Wang et al. (2015) atlas, these ROIs were manually drawn based on the results of a population receptive field (pRF) mapping condition that was included in the NSD protocol. The ROIs extended from the fovea (0° eccentricity) to peripheral cortical regions with reliable pRF signals (∼5°–6° eccentricity). We combined the ventral and dorsal regions of a given area by combining the ventral and dorsal ROIs (see below).

In our third set of NSD analyses, we evaluated two dorsal ROIs (Figure 2). A *mid‐parietal* ROI was drawn based on the union of V3A and V3B, which are sensitive to depth, motion, and structure as well as higher‐level visual‐spatial processing, respectively. Note that this ROI is separate from and dorsal to the V3 areas in the ventral stream (V3v and V3d; described above). In addition, a *superior parietal* ROI was drawn to border the union of IPS0, IPS1, IPS2, IPS3, IPS4, IPS5, and SPL1, which are involved in spatial attention, eye movements, visually guided action, and navigation. We did not evaluate the frontal eye fields, as this was not an ROI delineated in the NSD dataset, nor did we include other dorsal regions lacking well‐established topographic organization (e.g., OPA). Given that these large ROIs encompass many subregions, we also conducted a set of supplemental analyses disaggregating these ROIs using the Wang atlas (see Supporting Materials).

#### 2.4.3 Representational Similarity Matrices

We used RSA to relate the spatial distribution of fixations to the pattern of BOLD activation across voxels within each of the three fMRI ROIs. This approach has been used to understand aspects of visual processing in infancy (Bayet et al. 2020; Ellis 2024; Kiat et al. 2022; Spriet et al. 2022; Xie et al. 2022), but not yet to compare infants’ spatial distribution of fixations to the topographically mapped regions of visual cortex.

**FIGURE 3:**
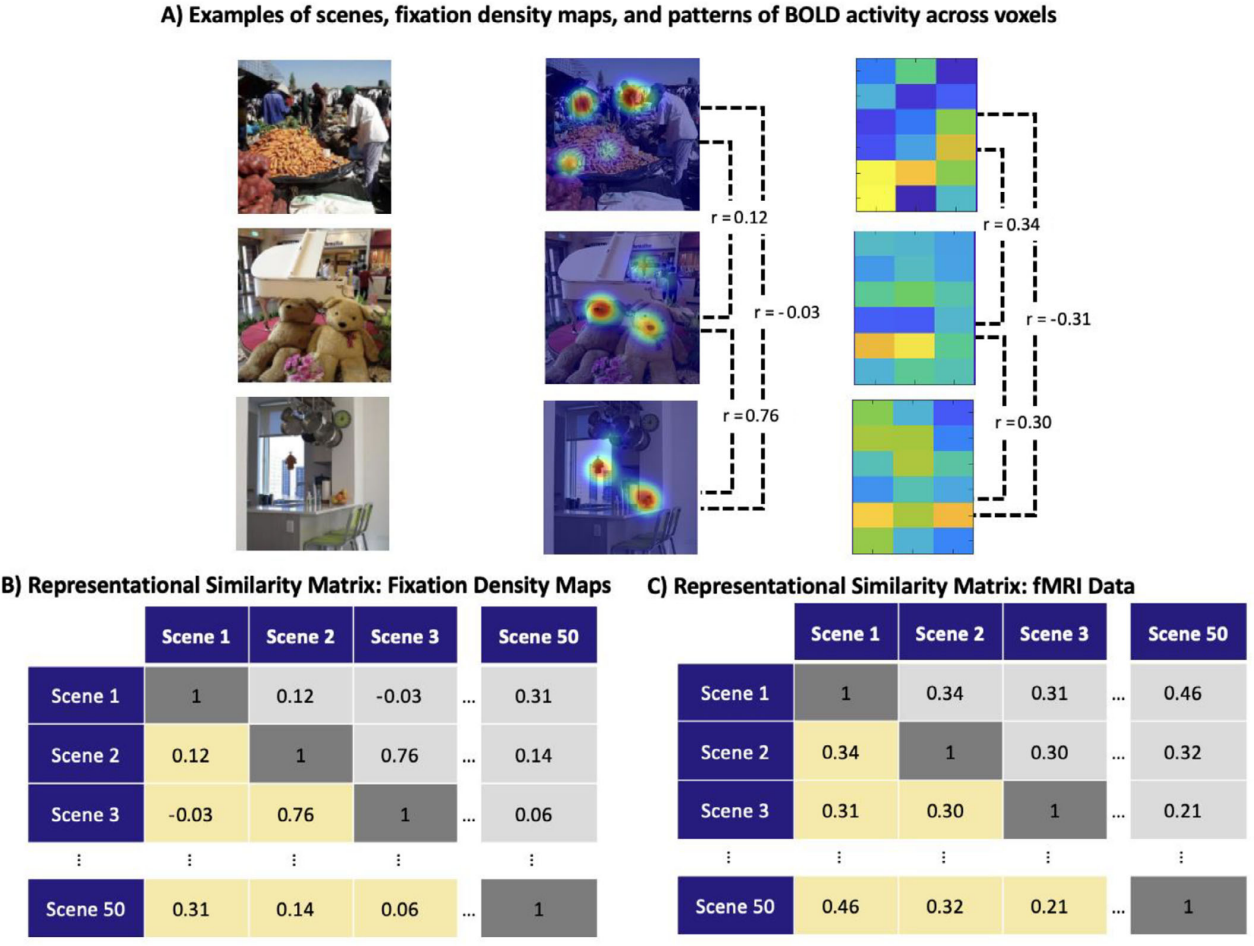
Overview of representational similarity analysis. We created (A) fixation density maps for each scene viewed by each participant and extracted the pattern of BOLD activity across voxels in each ROI in the NSD. We then correlated these fixation density maps to construct (B) RSMs based on fixation data. We similarly correlated the patterns of BOLD values across voxels within a given ROI to construct (c) RSMs based on adult fMRI data. Representational similarity is then computed as the (rank order) correlation between the fixation and fMRI RSMs. Note that the BOLD patterns shown here are illustrative rather than actual.

We first created *representational similarity matrices* (RSMs) for each infant's fixation data and for each adult's pattern of BOLD activation across voxels within a given ROI (see Figure  3A), which allowed us to compare data in different formats and units. To create the RSMs for infants’ fixation density maps, we first reorganized each 2D fixation density map into a single vector. For each pair of scenes viewed by a given infant, we computed the Pearson *r* correlation between the fixation density vectors to quantify the similarity of eye movement patterns. The *r* values for each pair of scenes viewed by that infant were then organized into an *N* × *N* RSM, where *N* was the number of scenes viewed by that infant (see Figure 3B). These RSMs quantify the extent to which the eye movement patterns were similar across pairs of scenes, which can be conceived as the *representational geometry* for the gaze data.

We similarly created RSMs based on each adult's fMRI activity, separately for each ROI. First, we organized the pattern of BOLD values across voxels within a given ROI into a single vector of activation values, separately for each image for a given participant. For analyses on early visual cortex (V1–V3), we appended the ventral and dorsal activation vectors (e.g., V1v and V1d) before creating RSMs. We computed the Pearson *r* correlation between the BOLD activation vectors for each pair of scenes to reflect the similarity of the neural response between those two scenes. The *r* values for the different scene pairs were then organized into an *N* × *N* RSM (as in Figure 3C). These RSMs quantify the extent to which the BOLD activation patterns were similar across pairs of scenes, which can be conceived as the representational geometry for a given cortical ROI. Note that we never averaged RSMs across fMRI participants, because this can misrepresent the representational geometry (Ashby et al. 1994).

The representational similarity between two datasets is defined as the similarity between the RSMs of the datasets (Kriegeskorte 2008). Here, representational similarity was quantified as the rank‐order correlation (i.e., Spearman *rho*) between each fixation RSM and each of the fMRI RSMs. Thus, this approach yielded 64 representational similarity estimates for each participant (one for each of eight ROIs for each of the eight adult fMRI subjects). We averaged the representational similarity estimates across the eight adult subjects, resulting in one representational similarity estimate for each ROI per participant. Note that, because the *rho* value was computed separately for each participant, the RSMs used to compute *rho* for a given participant included only the scenes that were viewed by that participant.

The representational similarity estimate is limited by the noise level of both the fixation density map and the adult fMRI data, which can be quantified with the *noise ceiling* of each dataset (Nili et al. 2014). To estimate the highest representational similarity (*rho*) that could be expected given the noise in both data sources, we took the product of the fixation and fMRI noise ceilings (see full details in Supporting Materials). The final noise ceiling estimates ranged from 0.10 to 0.30 for the lower bound and 0.22 to 0.36 for the upper bound, depending on the ROI and age group. Although *rho* values of these magnitudes would be difficult to detect in a traditional correlation between two simple measures, RSA provides the sensitivity needed to detect these small effects by calculating separate correlations from a large number of item pairs.

#### 2.4.4 Statistical Analyses

All analyses were conducted in R (R Core Team 2019). All analysis scripts and data files are available at https://osf.io/juw78/. We applied general linear mixed‐effect models (GLMM) using the mixed() function in the afex package (Singmann et al. 2016). For the infant sample, the representational similarity (Spearman *rho*) values served as the continuous dependent variable in mixed‐effects models with infant age (young: 5–7 months; old: 10–12 months) and ROI as categorical fixed effects, and a random intercept for subject. The model was specified in afex as:

$$\text{Spearman rho}\;∼{\mathrm{age}}^{*}\mathrm{ROI}+\left(1|\mathrm{subject}\right)$$

We wrapped the anova() function around the model to obtain *F*‐statistics and *p* values. We used the emmeans package (Lenth et al. 2023) to conduct post hoc *t*‐tests on estimated marginal means. A Holm–Bonferroni correction was applied to adjust for multiple comparisons across ROIs within each age group, controlling the family‐wise Type I error rate at 0.05. Adult data were intended for comparison purposes and are therefore not included in the same statistical models with infant data. In all adult analyses, we did not include the effect of age in the statistical models. Although infant and adult data were not analyzed in the same statistical models, we plotted them together below for ease of interpretation.

## 3 Results

Each scene was viewed by 17–37 participants per age group (*M_young_* = 23.86, SD_young_ = 3.34; *M_old_* = 23.73, SD_old_ = 3.42; *M_adult_* = 28.13, SD_old_ = 3.45). The basic looking characteristics are presented in Table 1. Independent samples *t*‐tests revealed that younger and older infants did not differ significantly in the number of trials completed, average number of fixations per trial, average fixation duration, or calibration accuracy (*p*’s > 0.30). However, compared to younger infants, older infants looked significantly longer per trial, *t*(91) = 4.97, *p* < 0.001. Adults had greater calibration accuracy, contributed more trials, made more fixations per trial, had longer average fixation durations, and looked longer to trials overall compared to both infant age groups (all *p*’s < 0.001).

**TABLE 1:** Mean looking characteristics (SD) for each sample.

	*N*	Trials with useable data (out of 50 max)	Fixations per trial	Average fixation duration (ms)	Average total looking duration (in milliseconds; out of 5000 ms)	Average calibration accuracy (°)
Young (5–7 months)	47	39.60 (9.45)	9.14 (1.92)	443.63 (70.87)	3042.34 (518.350)	0.80 (0.56)
Old (10–12 months)	46	40.24 (9.94)	9.50 (1.39)	435.13 (62.13)	3560.75 (485.71)	0.71 (0.42)
Adult	45	48.76 (4.82)	15.96 (1.49)	297.38 (39.61)	4273.58 (270.67)	0.49 (0.30)^a^

^a^ Calibration accuracy was missing for seven adult subjects.

### 3.1 Ventral Stream

#### 3.1.1 Low‐, Mid‐, and High‐Level ROIs

**FIGURE 4:**
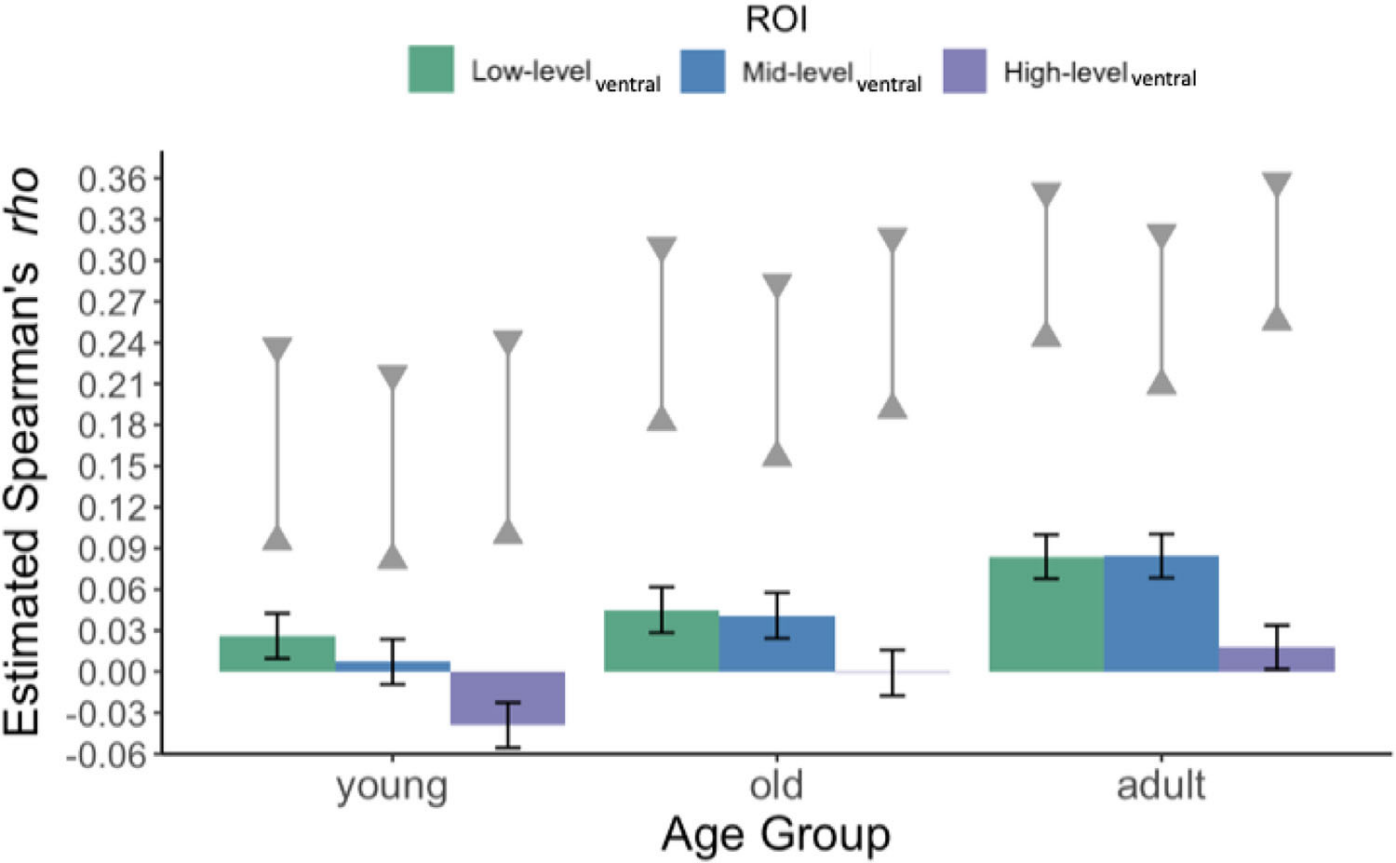
Estimated representational similarity (Spearman's *rho*) between fixation density maps and patterns of activation across ROIs in the adult fMRI data. Bars reflect mean values, and error bars reflect upper and lower 95% confidence intervals. The noise ceiling estimates (upper and lower triangles connected by a solid gray line) represent upper and lower bounds of the highest values that could be expected given the noise in both the fixation and fMRI data. Note that infant data (young, old) were analyzed in a separate statistical model from the adult data and are plotted here for visualization purposes.

Our first analysis evaluated representational similarity between fixations and fMRI ROIs along the ventral visual processing stream. Figure 4 shows the mean representational similarity estimates for each ROI in each age group, along with the noise ceiling for each age group. Note that these *rho* values are very small compared to the kinds of correlations usually observed in psychological research. However, small values like these in RSA can reflect very meaningful effects when viewed in relation to the *noise ceiling*, which is an estimate of the largest correlation that could be expected given the noise in the data. In the data shown in Figure 4, the *rho* values were quite substantial relative to the noise ceilings, suggesting a meaningful relationship between the representational geometry of the fixation data and the representational geometry of the adult fMRI data. In the younger infant age group, representational similarity was greatest for the low‐level visual cortex ROI and then at or below zero for mid‐ and high‐level areas. In the older infant age group and adults, representational similarity was approximately equal for low‐level_ventral_ and mid‐level_ventral_ ROIs but was near zero for the high‐level_ventral_ ROI.

The statistical analysis on infant data revealed significant main effects of ROI, *F*(2, 182) = 115.62, *p* < 0.001, and age group, *F*(1,91) = 7.63, *p* = 0.007. Importantly, there was a significant interaction between ROI and age, *F*(2, 182) = 3.40, *p* = 0.036.

In the younger infants, the *rho* values were significantly greater for the low‐level_ventral_ ROI than for the mid‐level_ventral_ ROI and high‐level_ventral_ ROI (Table 2). Additionally, among these younger infants, the *rho* values were significantly greater for the mid‐level_ventral_ ROI than the high‐level_ventral_ ROI. However, these *rho* values were at or below zero. Evaluation of the 95% confidence intervals for the estimated marginal means indicated that the *rho* values were significantly above chance (zero) only for the low‐level ROI in this age group, providing no evidence for representational similarity between fixations and adult fMRI data beyond the early visual cortex. Although the young infants’ *rho* values were below zero for the high‐level_ventral_ ROI, negative representational similarity values are not usually interpretable.

**TABLE 2:** Post hoc comparisons of estimated *rho* values between each ROI.

Age group	Comparison	*t*	*p*
		*df*_infants_ = 182, *df*_adults_ = 88	
**Low‐, mid‐, high‐level_ventral_ ROIs**			
Young (5–7 months)	Low‐level_ventral_ vs. mid‐level_ventral_	3.47	0.001^**^
	Low‐level_ventral_ vs. high‐level_ventral_	12.01	<0.001^***^
	Mid‐level_ventral_ vs. high‐level_ventral_	8.54	<0.001^***^
Old (10–12 months)	Low‐level_ventral_ vs. mid‐level_ventral_	0.75	0.458
	Low‐level_ventral_ vs. high‐level_ventral_	8.38	<0.001^***^
	Mid‐level_ventral_ vs. high‐level_ventral_	7.63	<0.001^***^
Adult	Low‐level_ventral_ vs. mid‐level_ventral_	−0.10	0.92
	Low‐level_ventral_ vs. high‐level_ventral_	11.19	<0.001^***^
	Mid‐level_ventral_ vs. high‐level_ventral_	11.29	<0.001^***^
**V1, V2, V3 ROIs**			
Young (5–7 months)	V1 vs. V2	3.95	0.001^**^
	V1 vs. V3	9.20	<0.001^***^
	V2 vs. V3	5.24	<0.001^***^
Old (10–12 months)	V1 vs. V2	0.25	0.807
	V1 vs. V3	2.46	0.044^*^
	V2 vs. V3	2.22	0.056
Adult	V1 vs. V2	−0.74	1.00
	V1 vs. V3	−0.34	1.00
	V2 vs. V3	0.40	1.00
**Dorsal ROIs**			
Young (5–7 months)	Mid‐parietal vs. superior parietal	3.95	0.001^**^
Old (10–12 months)	Mid‐parietal vs. superior parietal	0.25	0.807
Adult	Mid‐parietal vs. superior parietal	0.40	1.00

*** *p* < 0.001.

** *p* < 0.01.

* *p* < 0.05.

In the older infants, the representational similarity was nearly identical for the low‐level_ventral_ and mid‐level_ventral_ ROIs, which did not differ significantly from each other. However, these low‐ to mid‐level *rho* values were significantly greater than those from the high‐level_ventral_ ROI (see Table 2). The 95% confidence intervals for the estimated marginal means indicated that the representational similarity was significantly greater than chance (zero) for both the low‐ and mid‐level ROIs, but not for the high‐level ROI.

Comparison between the two infant age groups for each ROI revealed significantly greater representational similarity for older infants compared to younger infants for the mid‐level_ventral_ ROI, *t*(122) = 2.85, *p* = 0.005, but not the low‐level_ventral_ ROI, *t*(122) = 1.60, *p* = 0.111. The representational similarity was also significantly greater for older infants compared to younger infants for the high‐level_ventral_ ROI, *t*(122) = 3.23, *p* = 0.002. However, *rho* values were at or below zero for this ROI, suggesting that the spatial distribution of fixations was not linked to adults’ patterns of BOLD activity in this ROI for either age group.

Analyses of the adult sample revealed a significant main effect of ROI, *F*(2, 88) = 84.23, *p* < 0.001. The pattern of results mirrored those observed for older infants, except that the representational similarity values were higher in the adults (as would be expected given their higher noise ceiling). The representational similarity in adults was nearly identical for the low‐level_ventral_ and mid‐level_ventral_ ROIs, which did not differ significantly from each other. However, these low‐ to mid‐level *rho* values were significantly greater than those from the high‐level_ventral_ ROI (see Table 2). The 95% confidence intervals for the estimated marginal means indicated that adults’ representational similarity was greater than chance for all three ROIs. However, the effect for the high‐level_ventral_ ROI was weak, just slightly above chance, and was no longer significant when controlling for the role of physical salience (see Supporting Materials). Thus, except for this weak effect for the high‐level_ventral_ ROI, the pattern of results in adults was similar to that obtained for the older infants.

To assess the extent to which these results simply reflected attentional guidance by physical saliency, we recalculated the representational similarity between the fixation and fMRI RDMs, controlling for a saliency RDM generated from the Graph‐Based Visual Saliency model (Harel et al. 2007). Older infants and adults showed significant representational similarity for both the low‐level_ventral_ and mid‐level_ventral_ ROIs after the role of physical salience was regressed out. However, younger infants no longer showed significant representational similarity between their fixation patterns and the adult fMRI data in early visual cortex when salience was controlled for (see Supporting Materials). This is consistent with prior research indicating that physical salience plays a large role in guiding gaze in young infants (e.g., Kwon et al. 2016; Pomaranski et al. 2021).

#### 3.1.2 Disaggregated V1, V2, and V3 ROIs

**FIGURE 5:**
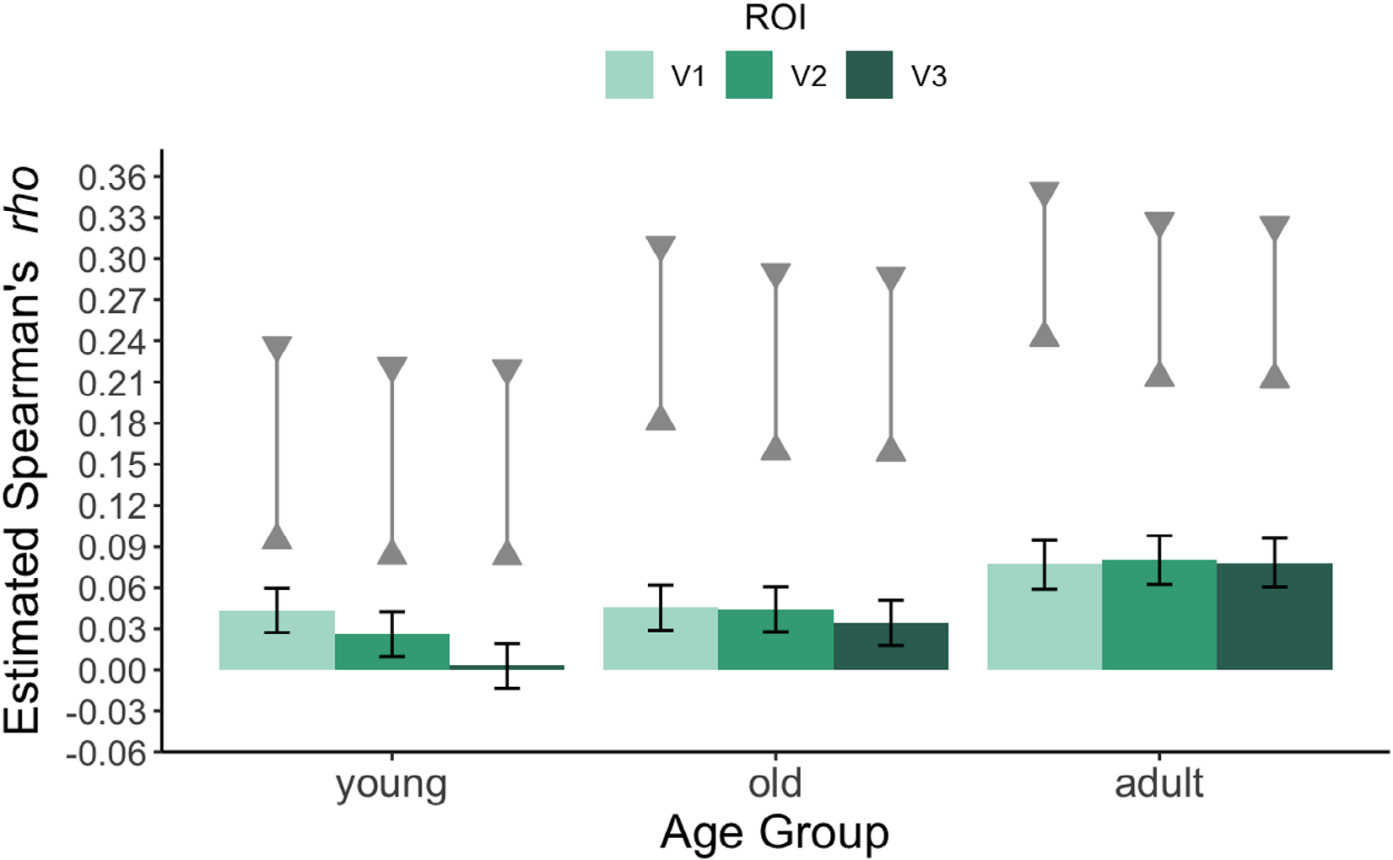
Estimated representational similarity (Spearman's *rho*) between fixation density maps and patterns of activation across ROIs in the adult fMRI data. Bars reflect mean values, and error bars reflect upper and lower 95% confidence intervals. The noise ceiling estimates (upper and lower triangles connected by a solid gray line) represent upper and lower bounds of the highest values that could be expected given the noise in both the fixation and fMRI data. Note that infant data (young, old) were analyzed in a separate statistical model from the adult data and are plotted here for visualization purposes.

In the prior analysis, all age groups demonstrated significant representational similarity with the low‐level ROI. However, this ROI was drawn to encompass a large area of the early visual cortex and does not allow for assessment of specific subregions. To address this, we next examined representational similarity between fixation patterns and separate V1, V2, and V3 ROIs. Figure 5 shows the mean representational similarity estimates for each ROI in each age group. In the younger infants, representational similarity was greatest for V1 and decreased to nearly zero by V3. In the older infant age group and adults, representational similarity was approximately equal across V1, V2, and V3.

The statistical analysis of the infant data revealed a significant main effect of ROI, *F*(2, 182) = 34.68, *p* < 0.001, and a significant interaction between ROI and age, *F*(2, 182) = 11.19, *p* < 0.001. However, the main effect of age group was not significant in this model, *F*(1,91) = 2.40, *p* = 0.125.

In the younger infants, the *rho* values were significantly greater for the V1 ROI than for the V2 and V3 ROIs, and greater for the V2 ROI than the V3 ROI (Table 2). Evaluation of the 95% confidence intervals for the estimated marginal means indicated that the *rho* values in this age group were significantly above chance (zero) for V1 and V2, but not V3.

In the older infants, the representational similarity was nearly identical for the V1 and V2 ROIs, which did not differ significantly from each other. Furthermore, the *rho* values for the V1 and V2 ROIs were slightly greater than those from the V3 ROI (see Table 2). Representational similarity for this age group was significantly greater than chance (zero) for V1, V2, and V3.

Comparison between the two infant age groups for each ROI revealed significantly greater representational similarity for older infants compared to younger infants for V3, *t*(111) = 2.69, *p* = 0.008, but not for V1, *t*(111) = 0.17, *p* = 0.869, or V2, *t*(111) = 1.56, *p* = 0.121.

Analyses of the adult sample revealed no significant main effect of ROI, *F*(2, 88) = 0.27, *p* = 0.761. The pattern of results was similar to that observed among older infants, with representational similarity significantly greater than chance (zero) for V1, V2, and V3.

### 3.2 Dorsal Stream ROIs

**FIGURE 6:**
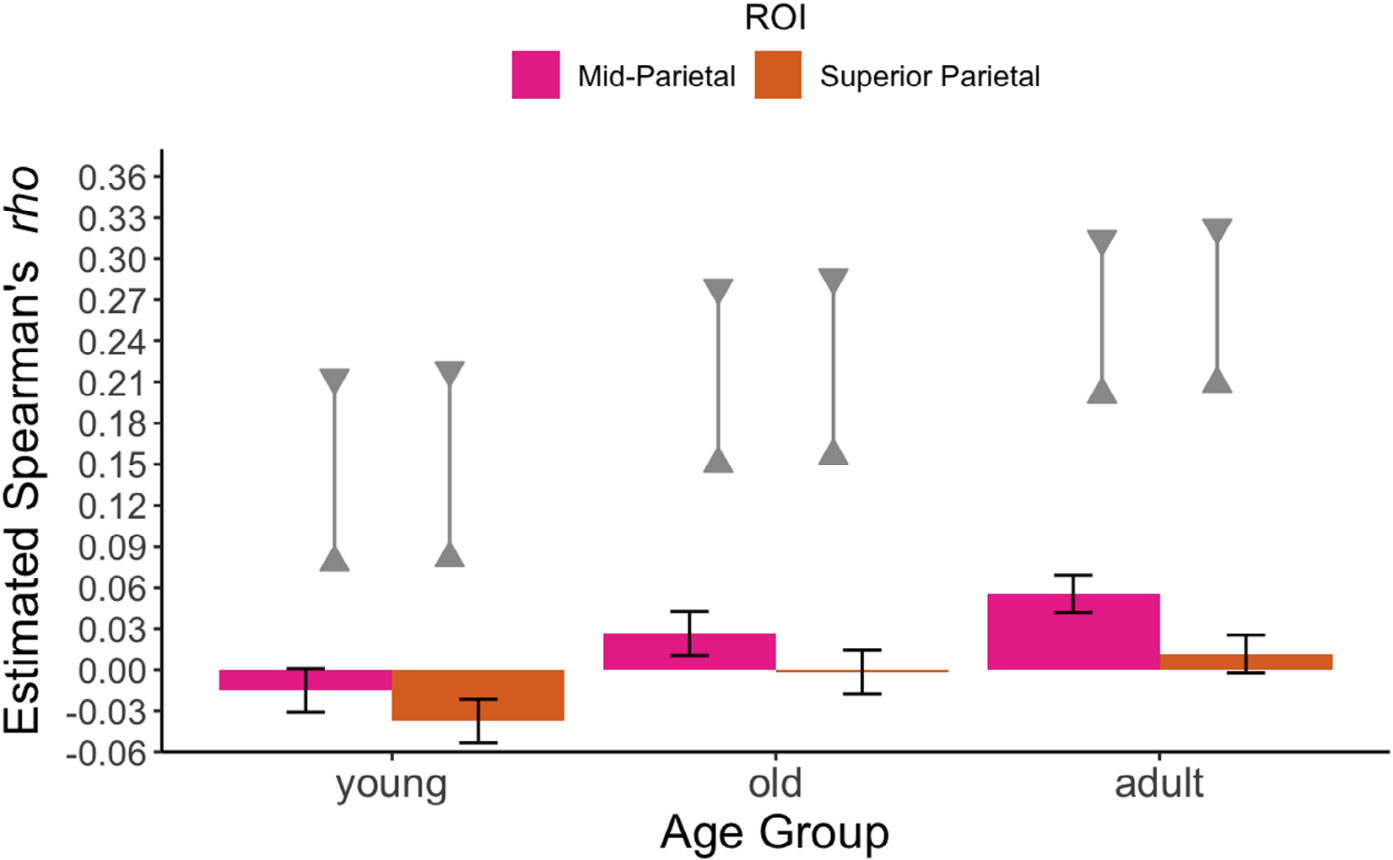
Estimated representational similarity (Spearman's *rho*) between fixation density maps and patterns of activation across ROIs in the adult fMRI data. Bars reflect mean values, and error bars reflect upper and lower 95% confidence intervals. The noise ceiling estimates (upper and lower triangles connected by a solid gray line) represent upper and lower bounds of the highest values that could be expected given the noise in both the fixation and fMRI data. Note that infant data (young, old) were analyzed in a separate statistical model from the adult data and are plotted here for visualization purposes.

Finally, we evaluated representational similarity between fixations and ROIs within two ROIs from the dorsal visual stream. Figure 6 shows the mean representational similarity estimates for each ROI in each age group, along with the noise ceiling for each age group. In the younger infant age group, representational similarity was at or below zero for both dorsal regions. In the older age group and adults, representational similarity was greater for the mid‐parietal ROI compared to the superior parietal ROI.

The statistical analysis of the infant data revealed a significant main effect of ROI, *F*(2, 182) = 75.44, *p* < 0.001, and age group, *F*(2, 182) = 12.28, *p* < 0.001. However, the interaction between ROI and age was not significant in this model, *F*(1,91) = 1.08, *p* = 0.302.

In the younger infants, the *rho* values were significantly greater for the mid‐parietal than for the superior parietal ROIs (Table 2). However, both of these *rho* values were at or below zero, so the difference between them is difficult to interpret and likely spurious. In the older infants, the representational similarity was also greater for the mid‐parietal ROI than for the superior parietal ROI. The 95% confidence intervals for the estimated marginal means indicated that the representational similarity was significantly greater than chance (zero) for the mid‐parietal ROI but not for the superior parietal ROI.

Comparison between the two infant age groups for each ROI revealed significantly greater representational similarity for older infants compared to younger infants for both the mid‐parietal ROI, *t*(91) = 5.43, *p* < 0.001, and the superior parietal ROI, *t*(91) = 6.84, *p* < 0.001.

Analyses of the adult sample revealed a significant main effect of ROI, *F*(1, 44) = 74.55, *p* < 0.001. The pattern of results was similar to that observed among older infants, such that representational similarity was significantly greater for the mid‐parietal ROI (which was above chance) than for the superior parietal ROI (which was not above chance).

As detailed in the Supporting Materials, disaggregating these ROIs further into their respective subregions yielded overall similar results: Younger infants did not show representational similarity for any ROI within these mid‐parietal and superior parietal regions. When the mid‐parietal ROI was subdivided, older infants did not demonstrate representational similarity for either V3A or V3B, nor any subregion within the superior parietal ROI. Adults demonstrated representational similarity for both ROIs within the mid‐parietal region, as well as several regions within the superior parietal region (IPS0, IPS1, IPS4, SPL1).

## 4 Discussion

We related the pattern of infant and adult eye movements to the patterns of activation in regions across adults’ ventral visual processing pathways. We found significant age‐related differences across the analyses, with gaze patterns predicted by higher‐level regions in older infants and adults. Specifically, younger infants’ fixations were significantly predicted only by low‐level areas (V1, V2) of the ventral stream, whereas fixations of older infants and adults were predicted by both low‐ and mid‐level regions of the ventral stream and also by mid‐level regions of the dorsal stream. These results are consistent with the hypothesis that gaze becomes controlled by increasingly abstract representations across the first year of postnatal life (Johnson 1990). More specifically, these results suggest that where older infants look is controlled by more sophisticated representations like those found in mid‐level regions of the adult ventral processing stream.

Although we did not find significant representational similarity between older infants’ spatial distribution of fixations and the high‐level_ventral_ ROI, this does not imply that older infants do not also use high‐level abstract representations to guide their looking. The high‐level_ventral_ ROI in our analyses encompassed a large area of visual cortex that may be insensitive to the topographic mapping of features, making spatial fixation metrics less sensitive to the patterns of activity in these areas. Future work using metrics that are not spatially defined (e.g., overall looking time) may reveal an influence of the high‐level_ventral_ ROI. It is also possible that the lack of representational similarity for the high‐level_ventral_ ROI is a result of the heterogeneity of the areas that were combined into this ROI.

The older infants’ fixations were equally consistent with low‐level_ventral_ and mid‐level_ventral_ cortical regions. This finding contrasts with the finding of Kiat et al. (2022) that early layers of AlexNet predicted younger infants’ fixations to a greater extent than older infants and with the finding of Pomaranski et al. (2021) that salience models have a decreasing ability to predict fixations among older infants. One possibility for the differences between the current results and those reported by Kiat et al. (2022) and Pomaranski et al. (2021) is that these previous studies used identical scenes and participants. In the current study, we used a novel stimulus set; these new images may have elicited overall greater activation in low‐level visual cortex and early layers of AlexNet, leading older infants’ fixations to continue demonstrating looking patterns consistent with early visual processing. It is also possible that low‐level areas of the ventral stream may continue to have a strong role in guiding fixations across development, even when infants’ fixations can be guided by mid‐ or high‐level ventral stream areas, and this effect simply was not detected in the prior studies. This interpretation is supported by the adult data, which also demonstrated similar effects for the low‐ and mid‐level ventral stream ROIs.

Our findings converge with contemporary hypotheses proposing that the development of visual attention is not merely a shift from low‐ to high‐level processing but rather a more complex integration of both processes (Lynn et al. 2024; Markant and Scott 2018; Oakes 2023). The persistent influence of early visual areas may reflect a mechanism that ensures that infants remain highly responsive to basic visual features as they learn to interpret more complex scenes using increasingly abstract features, such as those coded by the adult mid‐level visual cortex. Our findings are also consistent with the contemporary view of adult visual attention (e.g., Todd and Manaligod 2018), such that stimulus features beyond low‐level salience may guide where infants look.

Finally, this study was the first to explore infants’ gaze patterns to neural representations in the dorsal visual stream. We found weak but nonetheless significant representational similarity between the mid‐parietal regions and the fixations of both older infants’ and adults’, but not those of younger infants. We speculate that these effects were small because participants in the NSD study were required to keep their gaze fixed at the center of the display while viewing the scenes, likely reducing dorsal stream activity related to shifts of overt attention. Future research should further investigate how dorsal regions support gaze control in infancy, and how the onset of self‐initiated locomotion (e.g., crawling, walking) may contribute to the functional development of these areas.

More broadly, our findings suggest that higher‐order ventral visual areas may play an increasing role in guiding visual attention across infancy, reflecting a developmental shift toward a more abstract representation of the environment. Future work should evaluate how these developments relate to higher‐order cognitive functions such as recognition and navigation.

## Author Contributions

**Brianna K. Hunter**: conceptualization, data curation, formal analysis, visualization, writing – original draft, methodology, funding acquisition, investigation. **John E. Kiat**: writing – review and editing, methodology, data curation, conceptualization. **Steven J. Luck**: conceptualization, methodology, writing – review and editing, supervision, data curation, funding acquisition, resources. **Lisa M. Oakes**: conceptualization, data curation, methodology, supervision, writing – review and editing, funding acquisition, resources.

## Conflicts of Interest

The authors declare no conflicts of interest.

## Supporting information




**Supporting File 1**: desc70076‐sup‐0001‐SuppMat.docx

## Data Availability

The data that support the findings of this study are openly available in OSF at https://osf.io/juw78.
