# Supplementary material for: Relating Infant Fixations to Adult Cortical Activation Patterns Using the Natural Scenes Dataset
Source: Dev Sci. 2025 Sep 19;28(6):e70076. doi: 10.1111/desc.70076 (PMC12447543; doi:10.1111/desc.70076)
Supplement: Supplementary file 1 — Supporting File 1: desc70076‐sup‐0001‐SuppMat.docx [file DESC-28-e70076-s001.docx]

Supplementary Materials for

**Relating Infant Fixations to Adult Cortical Activation Patterns using the Natural Scenes Dataset**

Brianna K. Hunter

Contents

S1. Noise Ceiling Estimates

S2. Partialling out the Role of Salience

S3. Disaggregating Dorsal Stream ROIs

**S1. Noise Ceiling Estimates**

To quantify the noise ceilings for these datasets, we used the general approach of Nili et al. (2014). Because each participant viewed a different number of scenes, we were unable to compute the true upper and lower limits of the fixation noise ceilings, but we were able to estimate them. To do this, we padded each participants’ RSM with NaNs for the scene pairs they did not view, producing a 78 x 78 RSM for each infant (where 78 is the total number of scenes across all participants). We then created an average RSM for each age group using these padded RSMs (excluding the NaN values) and correlated each participant’s padded RSM (again excluding all NaN values) to this average matrix. We then averaged the resulting correlation values across the participants of a given age group to estimate the upper bound of the noise ceiling. To estimate the lower bound, we repeated the same procedures but instead of correlating individual RSMs to a grand average RSM, we created average RSMs for each participant that included all other participants of the same age (i.e., leaving one participant out of each average RSM). We then averaged the resulting correlation values across the participants of a given age group. See Table S1 for upper- and lower-bound estimates.

We calculated the upper-bound noise ceiling for the fMRI data by correlating each adult subject’s RSM to the average RSM across all subjects for each ROI, and the lower-bound noise ceiling by using the leave-one-out approach described above [(Nili et al., 2014)](https://www.zotero.org/google-docs/?mVJom7). The noise ceilings for the fMRI data are in Table S1.

The correlation between the fixation RSMs and the fMRI RSMs was limited by the product of the noise ceilings for the two RSMs. Thus, to estimate the highest representational similarity (*rho*) that could be expected given the noise in both the fixation and adult fMRI data, we took the product of these two noise ceilings. The final infant lower-bound noise ceiling estimates (fixation noise ceiling X adult fMRI noise ceiling) ranged from .10 to .30 and the upper-bound estimates ranged from .21 to .36, depending on the ROI and age of participant.

*Table S1. Lower- and upper-bound noise ceiling estimates*

|  | Lower-bound | Upper-Bound |
| --- | --- | --- |
| **Fixation Data** |  |  |
| Young (5-7 months) | .18 | .36 |
| Old (10-12 months | .34 | .47 |
| Adults | .45 | .53 |
| **fMRI Data** |  |  |
| Low-Level_ventral_ | .54 | .66 |
| V1 | .54 | .66 |
| V2 | .47 | .62 |
| V3 | .47 | .61 |
| Mid-Level_ventral_ | .46 | .61 |
| High-Level_ventral_ | .57 | .68 |
| Mid-Parietal | .45 | .59 |
| V3A* | .42 | .57 |
| V3B* | .49 | .62 |
| Superior Parietal | .45 | .61 |
| IPS0* | .43 | .59 |
| IPS1* | .36 | .54 |
| ISP2* | .34 | .52 |
| IPS3* | .32 | .51 |
| IPS4* | .17 | .42 |
| IPS5* | .11 | .38 |
| SPL1* | .20 | .44 |

*Note:* * indicates ROIs presented in Supplemental only

**S2. Partialling out the Role of Salience**

Because low-level visual properties (e.g., contrast, color, orientation) may influence both fixations and fMRI responses, we conducted an additional analysis controlling for their shared variance using partial correlation. In this supplementary analysis, we recalculated representational similarity by computing the *partial* Spearman correlation between each fixation RDM and each fMRI RDM (one per fMRI subject), controlling for a saliency RDM generated from the Graph-Based Visual Saliency (GBVS) model (Harel et al., 2007). Partial correlations were computed by residualizing both the fixation and fMRI RDM vectors with respect to the GBVS RDM vector, followed by computing a Pearson correlation on the resulting residuals. This allowed us to assess whether spatial patterns of attention were related to adult fMRI responses above and beyond physical saliency.

Similar to the results reported in the main text, in the infant statistical model, we observed significant main effects of ROI, *F*(2, 182) = 74.49, *p* < .001, and age group, *F*(1,91) = 8.70, *p*  = .004. Importantly, there was again a significant interaction between ROI and age, *F*(2, 182) = 4.87, *p*  = .009. Figure S1 shows the mean partial representational similarity estimates for each ROI in each age group.

In the younger infants, the partial *rho* values were significantly greater for the low-level_ventral_ ROI than for the mid-level_ventral_ ROI, *t*(182) = 3.03, *p* = .003, and high-level_ventral_ ROI, *t*(182) = 10.55, *p* < .001. Additionally, among these younger infants, the *rho* values were significantly greater for the mid-level_ventral_ ROI than the high-level_ventral_ ROI, *t*(182) = 7.52, *p* < .001. However, in contrast to the findings reported in the main text, all partial *rho* values were at or below zero, providing no evidence for representational similarity between fixations in the younger infants and adult fMRI data.

Similar to the findings reported in the main text, the partial representational similarity among older infants was nearly identical for the low-level_ventral_ and mid-level_ventral_ ROIs, which did not differ significantly from each other, *t*(182) = 0.06, *p* = .950. These low- to mid-level *rho* values were significantly greater than those from the high-level_ventral_ ROI, *t_low_*(182) = 6.18, *p* < .001, *t_mid_*(182) = 6.24, *p* < .001. The 95% confidence intervals for the estimated marginal means indicated that the representational similarity was significantly greater than chance (zero) for both the low- and mid-level ROIs, but not for the high-level ROI.

In the adult statistical analysis, we again observed a significant main effect of ROI, *F*(2, 88) = 51.95, *p* < .00. The partial representational similarity among adults was nearly identical for the low-level_ventral_ and mid-level_ventral_ ROIs, which did not differ significantly from each other, *t*(188) = -1.44, *p* = .15. These low- to mid-level *rho* values were significantly greater than those from the high-level_ventral_ ROI, *t_low_*(88) = 8.02, *p* < .001, *t_mid_*(88) = 9.46, *p* < .001. The 95% confidence intervals for the estimated marginal means indicated that the representational similarity was significantly greater than chance (zero) for both the low-level_ventral_ and mid-level_ventral_ ROIs, but not for the high-level_ventral_ ROI. This pattern of results is consistent with older infants.


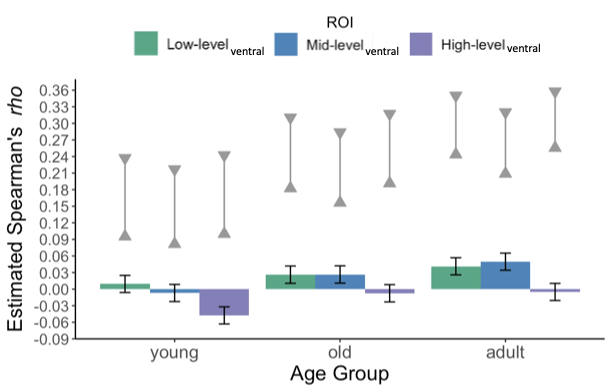


Figure S1. Estimated partial representational similarity (Spearman’s *rho*) between infant (left) and adult (right) fixation density maps and patterns of activation across ROIs in the adult fMRI data, partialling out the role of GBVS. Bars reflect mean values and error bars reflect upper and lower 95% confidence intervals. The noise ceiling estimates (upper and lower triangles connected by a solid gray line) represent upper and lower bounds of the highest values that could be expected given the noise in both the fixation and fMRI data. Note that infant data (young, old) were analyzed in a separate statistical model from the adult data and are plotted here for visualization purposes.

**S3. Disaggregating Dorsal Stream ROIs**

Our overall goal was to compare representational similarity across regions that differ in level of abstraction from low-level, early visual features to more complex, mid- and high-level features. We therefore used the pre-defined ROIs in the NSD, which represent large-scale stages of the visual processing hierarchy, rather than drawing new ROIs ourselves to assess individual subregions. However, we acknowledge that aggregating multiple areas into a single ROI may introduce additional variance into the RDMs. We therefore used the Wang atlas to calculate ROIs for subregions within both the mid-parietal (i.e., V3A, V3B) and superior parietal (i.e., IPS0, IPS1, IPS2, IPS3, IPS4, IPS5, and SPL1) regions. We followed the same representational similarity analysis approach described in the main text to relate adult activity within these ROIs to our participants’ fixation density maps.

Figure S2 shows the mean representational similarity estimates for each ROI in each age group. In the infant statistical model, we observed significant main effects of ROI, *F*(8, 728) = 10.77, *p* < .001, and age group, *F*(1,91) = 10.11, *p*  < .001. There was also a significant interaction between ROI and age, *F*(8, 728) = 5.36, *p*  < .001.

Among both the younger and older infant age groups, all *rho* values were not significantly above zero, indicating no representational similarity at any subregion. However, there were some significant differences in *rho* values across the ROIs for each age group. In the younger infants, *rho* was significantly lower for IPS0, IPS1, IPS2, and IPS3 compared to IPS4 and IPS5 (all *p*’s < .001). Furthermore, *rho* was significantly lower for IPS0 compared to SPL1 (*p* = .011) but IPS4 was significantly greater than SPL1 (p = .031). There were no other significant differences among younger infants. In the older infants, *rho* values for IPS2 and IPS3 were significantly lower than those for IPS4 (*p*’s < .010), but there were no other significant differences across the ROIs among older infants. The representational similarity among younger infants was lower than older infants for both mid-parietal ROIs (i.e., V3A and V3B, *p*’s < .001), as well as for IPS0 (*p* = .001), IPS1 (*p*  = .002), IPS2 (*p* = .004), IPS3 (*p* = .029) and SPL1 (*p* = .029). However, rho values were not significantly different among older vs younger infants for IPS4 (*p* = .063) or IPS5 (*p* = .280).

In the adult statistical model, we observed a significant main effect of ROI, *F*(8, 352) = 18.69, *p* < .001. Figure S2 shows the mean representational similarity estimates for each ROI. Adult *rho* values were significantly above zero for V3A, V3B, IPS0, IPS1, IPS4, and SPL1, indicating representational similarity at both mid-parietal subregions, and approximately half of the superior parietal regions.

Comparison of estimated marginal means revealed that *rho* values for the adults were significantly greater for both IPS0 and IPS1 compared to IPS2, IPS3, and IPS5 (all *p*’s < .030). Furthermore, *rho* was significantly greater for IPS2 compared to IPS4 (*p* < .001) and greater for SPL1 compared to IPS3 (p < .001). There were no other significant differences among adults.


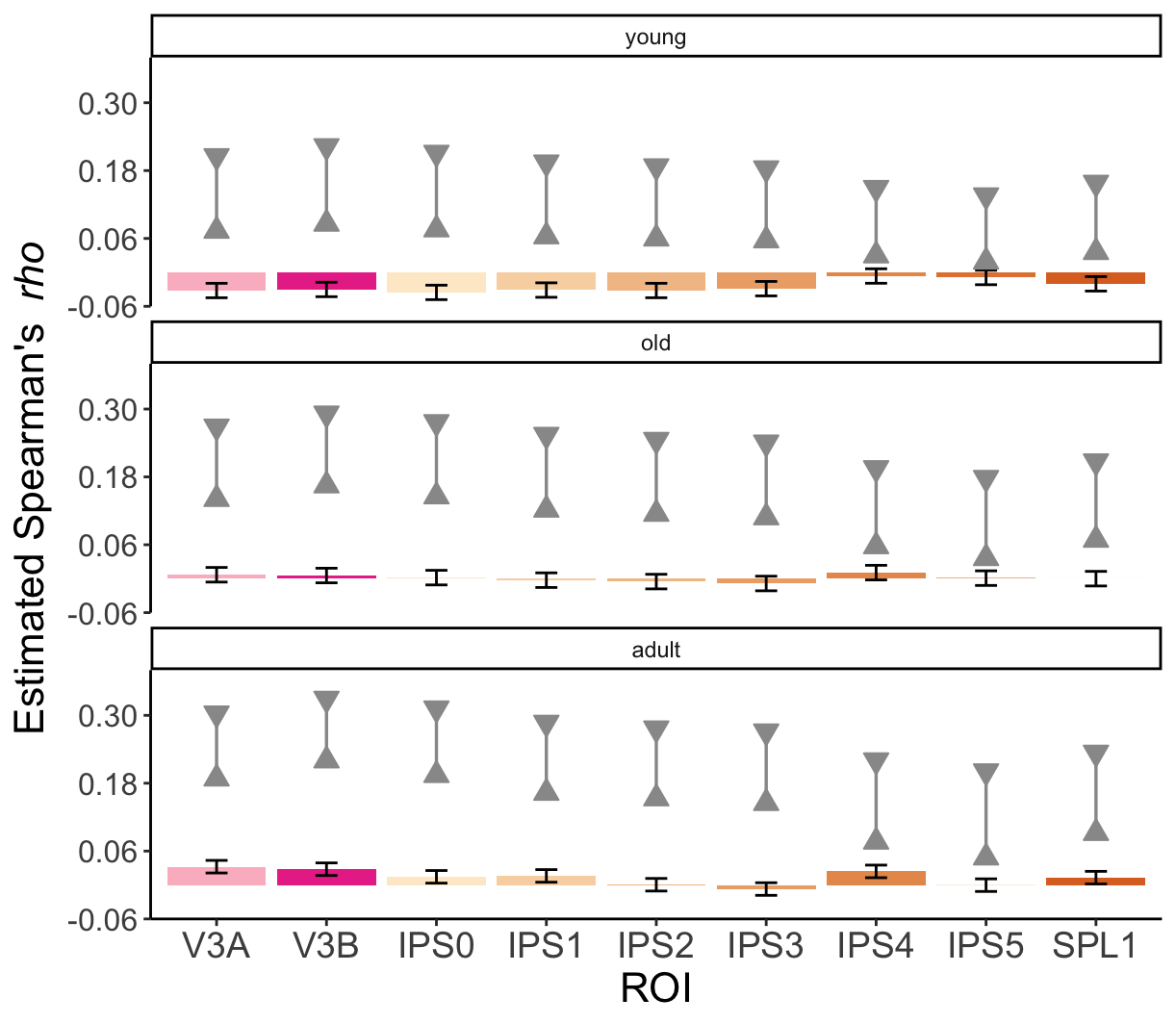


Figure S2. Estimated partial representational similarity (Spearman’s *rho*) between infant (bottom) and adult (top) fixation density maps and patterns of activation across ROIs in the adult fMRI data. Bars reflect mean values and error bars reflect upper and lower 95% confidence intervals. The noise ceiling estimates (upper and lower triangles connected by a solid gray line) represent upper and lower bounds of the highest values that could be expected given the noise in both the fixation and fMRI data. Note that infant data (young, old) were analyzed in a separate statistical model from the adult data and are plotted here for visualization purposes.
